# New immunotherapy strategies for patients with sarcomas: highlights from the 2023 ASCO annual meeting

**DOI:** 10.1186/s13045-023-01486-6

**Published:** 2023-08-08

**Authors:** Matthieu Roulleaux-Dugage, Antoine Italiano

**Affiliations:** 1 Department of Medicine, Institut Bergonié, 229 Cours de l’Argonne, 33000 Bordeaux, France; 2 DITEP, Gustave Roussy, Villejuif, France; 3 Faculty of Medicine, University of Bordeaux, Bordeaux, France

## Abstract

Immunotherapy has revolutionized cancer treatment, but currently, immuno-oncology agents have not been approved for patients with soft tissue sarcomas. However, there is growing evidence suggesting that immunotherapy could be an effective therapeutic strategy for this group of diseases. Here, we reviewed the latest advances of immunotherapy trials from the 2023 American Society of Clinical Oncology Annual Meeting, including some novel and encouraging combination regimens. Further research is still needed to fully understand the optimal use of these agents in sarcoma treatment.

Considering the recent advancements in immune checkpoint inhibitors (ICIs), there remains limited and controversial human data supporting the effectiveness of immunotherapy in soft tissue sarcomas (STS) based on the current clinical trials.

A recent analysis of pooled data from nine clinical trials investigating ICIs in sarcomas, comprising 384 patients, revealed an overall objective response rate (ORR) of 15.1% [1]. However, when excluding alveolar soft-part sarcoma, a rare subtype highly responsive to PD-1/PD-L1 monoclonal antibodies, the ORR dropped to 9.8% [1].

During the latest American Society of Clinical Oncology (ASCO) meeting, several studies presented combination therapeutic strategies which aimed at improving response rates to ICIs (Table 1). Three studies explored the combination of ICI with different chemotherapy regimens.

**Table 1:** Clinical trials investigating immunotherapy-based regimens in patients with advanced soft-tissue sarcomas (ASCO 2023)

Reference	Population	Line	Phase	Intervention	*n*	ORR—n (%)	mPFS (months)	mOS (months)	Safety results
7	LMS	First line	Ib	Doxorubicin 75mg/m^2^ D1 + DTIC 400mg/m^2^ D1-D2 + Nivolumab (240mg/360mg) D2 q3w for 6 cycles Followed by Nivolumab q3w 1 year as maintenance	16	9 (56.2%)	8.7	–	20% G3 Anemia 30% G3-4 Neutropenia 5% (*n* = 1) G3 Febrile neutropenia
5	All	First or Second line (no prior anthracycline/ICB)	II	Balsitilimab (αPD-1) 300mg q3w for 2 years Zalifrelimab (αCTLA-4) 1mg/kg q6w 4 cycles Doxorubicin 75mg/m^2^ q3w 6 cycles start at C1 (stage 2, n = 14) or C2 (stage 1, n = 19)	33	10 (33.3%)	5.6	14.5	12% G3 + irAE (2 colitis)
13	LMS	First or Second line (no prior anthracycline)	Ib	TTI-621 (αCD47 IgG1) 0.2mg/kg D1, D8—q3w Doxorubicin 75mg/m^2^ q3w up to 6 cycles	23	5 (21.7%)	–	–	8.7% G3 + Anemia 43.5% G3 + Neutropenia 4.3% (*n* = 1) G3 + Febrile neutropenia 4.3% (*n* = 1) G3 + pancreatitis
10	All	Second line and beyond	II	Arm A: cabozantinib 60 mg daily vs Arm B: Cabozantinib 40 mg daily + Ipililumab (1mg/kg) + Nivolumab (3 mg/jg) q3w for 4 cycles Followed by Cabozantinib 40 mg daily plus Nivolumab 480 mg q4w 1 year as maintenance	Arm A: 36 Arm B: 69	Arm A: 2 (6%) Arm B: 7 (11%)	Arm A: 3.75 Arm B: 5.36	Arm A: 22.6 Arm B: not reached	15.9% G3 Liver enzymes increase
8	Angiosarcomas	Previously treated with taxanes (ICB and VEGFR-TKI naive)	II	Nivolumab 480mg q4w Cabozantinib 40mg daily	22	13 (59.0%)	9.6	NR	36.4% G3 + TRAE

Reichardt et al. reported the findings of the NITRASARC study, which investigated the safety and efficacy of a regimen combining the cytotoxic agent trabectedin and the PD1 antagonist nivolumab in patients with advanced STS [2]. The study rationale was based on preclinical data suggesting that trabectedin could enhance the activity of immune-modulating agents by influencing the tumor microenvironment and reducing tumor-associated macrophages [3]. Trabectedin has shown synergistic effects with PD1 inhibition in preclinical models [4]. Forty-three patients with L-sarcomas and 49 patients with other sarcomas were enrolled. Safety was manageable, with the most frequent grade 3/4 adverse events being leucopenia, anemia, nausea, vomiting, and increased liver enzymes. The overall response rate was 10.9% (10% for cohort A and 13% for cohort B). Median progression-free survival (PFS) and overall survival (OS) for cohort A were 5.5 months (95% CI 2.2–11.3) and 18.7 months (95% CI 13.7–31.3), respectively. For cohort B, median PFS and OS were 2.3 months (95% CI 1.8–2.6) and 5.6 months (95% CI 3.5–8.6), respectively. However, the observed activity in this study did not surpass the expected outcomes with trabectedin alone.

Wilky et al. presented the results of a phase 2 study that investigated the combination of doxorubicin with zalifrelimab (a CTLA-4 inhibitor) and balstilimab (a PD1 inhibitor) in patients with advanced STS [5]. Doxorubicin has been shown to induce immunogenic cell death (ICD), triggering an immune response against dead-cell antigens and facilitating tumor antigen presentation and T-cell activation [6]. The study hypothesized that combining doxorubicin with zalifrelimab and balstilimab would improve the 6-month progression-free survival (PFS) rate compared to historical doxorubicin. Thirty patients with various STS types were enrolled, and four patients (12%) experienced grade 3/4 immune-related adverse events (colitis, pancreatitis, diabetic ketoacidosis, hypertriglyceridemia, hypothyroidism). The 6-month non-progression rate was 46.4% (95% CI 28–66), failing to reach the study’s objective of 63%.

Martin-Broto et al. reported the efficacy and safety data of a combination of nivolumab with the doxorubicin/dacarbazine chemotherapy regimen [7]. The rationale for this study was like the one reported by Wilky et al., based on doxorubicin’s potential to induce immunogenic cell death and synergize with PD1 targeting [6]. Thirty-six patients with advanced leiomyosarcomas were included, with 16 patients evaluable for efficacy. Safety was acceptable, with 15% of patients experiencing grade 3/4 neutropenia. Nine patients achieved an objective response, six had stable disease, and one had progressive disease. The median PFS was 8.7 months (95% CI 7.9–9.3).

In addition to chemotherapy combinations, studies have reported safety and efficacy data for combining ICIs with tyrosine kinase inhibitors. Grilley-Olson et al. presented the results of the Alliance A091902 study, a phase 2 study investigating the combination of nivolumab with cabozantinib in patients with advanced angiosarcomas who had previously failed taxane-based chemotherapy [8]. Previous evidence suggested that angiosarcomas might be more responsive to immune checkpoint inhibition [9]. Targeting angiogenesis with a multi-tyrosine kinase inhibitor and an ICI represented therefore a promising strategy for this subtype. Twenty patients participated in the study, and the combination was well-tolerated, with the most frequent treatment-related adverse event being hypertension. The objective response rate was 59%, with durable responses and a median PFS of 9.6 months (5.4-NR).

Cabozantinib was also combined with nivolumab and ipilimumab in a randomized phase 2 study comparing this combination to cabozantinib alone [10]. Sixty-nine patients were randomized to the combination arm, and 36 received cabozantinib monotherapy. The combination arm observed seven objective responses (11%), while the monotherapy arm had two (6%). The median PFS was 5.3 months (95% CI 4.1–11) for the combination and 3.5 months (95% CI 1.1–7.7) for monotherapy (*p* = 0.016). The median OS was 22.6 months (95% CI 14.8-NA) for the combination and not reached (9.6-NA) for monotherapy (*p* = 0.42). Notably, among the 19 patients from the cabozantinib monotherapy arm who were allowed to cross over to the combination arm, seven showed tumor shrinkage, suggesting at least an additive effect of the combination with nivolumab and ipilimumab.

Apart from immune checkpoint inhibition, other immune targets hold therapeutic potential for sarcomas. STS often exhibit heavy infiltration by tumor-associated macrophages (TAM) [11]. Additionally, STS, including leiomyosarcomas, express high levels of CD47, which suppresses phagocytic activity and enables tumor cells to evade immune-mediated clearance [12]. Movva et al. presented the results of a phase 2 study investigating TTI-621 in combination with doxorubicin in patients with advanced leiomyosarcomas [13]. Doxorubicin has been shown to promote tumor cell phagocytosis by inducing preapoptotic translocation of calreticulin to the cell surface [14]. Preclinical studies demonstrated enhanced anti-tumor activity and increased macrophage-mediated killing with the combination of doxorubicin and CD47-targeted antibodies [15]. TTI-621 is a recombinant soluble fusion protein that combines the Fc region of human IgG1 with the CD47 binding domain of human SIRPα, overriding CD47-mediated inhibition of phagocytosis and providing pro-phagocytic stimulation. Twenty-three patients were enrolled in the study, and the most frequent treatment emergent adverse events included neutropenia, anemia, and thrombocytopenia. Notably, five patients (21.7%) achieved an objective response.

It is crucial to emphasize that all these studies included patients without utilizing any biomarker-based selection strategy. While certain histological subtypes, such as undifferentiated pleomorphic sarcomas or de-differentiated liposarcomas, have shown anecdotal responses and potential sensitivity to treatment [9, 16], the current biomarker for immunotherapy in several epithelial tumors, PD-L1 expression, is not relevant for STS. Although PD-L1 expression of ≥ 1% in tumor cells was observed in over 15% of patients, it did not demonstrate a clear correlation with clinical benefits [1]. Furthermore, the tumor mutation burden was generally low across all histological subtypes, with a median burden of fewer than two mutations per megabase [17].

Recent data have suggested that the presence of tertiary lymphoid structures (TLS) may serve as a relevant strategy to identify patients with advanced STS who are more likely to benefit from immune checkpoint inhibition [18]. However, TLS are present in only 20% of STS cases. Therefore, investigating combination strategies to convert “cold tumors” into “hot tumors” and enhance their sensitivity to ICIs is of paramount importance.

The studies presented at ASCO explored combinations of immune-oncology agents with various drugs, including cytotoxic agents and VEGFR inhibitors. However, the main limitation of these studies is that almost all of them were single-arm and/or did not include analysis of sequential blood or tissue samples. Except for the study investigating the combination of nivolumab plus cabozantinib in angiosarcomas [8], the response and survival rates observed were similar to those seen with the current standard of care. Unfortunately, the absence of sequential tumor biopsies and randomization hinders drawing definitive conclusions regarding the influence of these combinations on the tumor microenvironment and their potential correlation with clinical benefits.
